# Human amyloid-β enriched extracts: evaluation of in vitro and in vivo internalization and molecular characterization

**DOI:** 10.1186/s13195-019-0513-0

**Published:** 2019-06-29

**Authors:** Cristina M. Pedrero-Prieto, Alicia Flores-Cuadrado, Daniel Saiz-Sánchez, Isabel Úbeda-Bañón, Javier Frontiñán-Rubio, Francisco J. Alcaín, Lourdes Mateos-Hernández, José de la Fuente, Mario Durán-Prado, Margarita Villar, Alino Martínez-Marcos, Juan R. Peinado

**Affiliations:** 1 Department of Medical Sciences, Ciudad Real Medical School, Oxidative Stress and Neurodegeneration Group, Regional Center for Biomedical Research, University of Castilla-La Mancha, Ciudad Real, Spain; 2 Department of Medical Sciences, Ciudad Real Medical School; Neuroplasticity and Neurodegeneration Group, Regional Center for Biomedical Research, University of Castilla-La Mancha, Ciudad Real, Spain; 3 SaBio. Instituto de Investigación en Recursos Cinegéticos IREC, CSIC-UCLM-JCCM, Ciudad Real, Spain; 4 UMR BIPAR, INRA, ANSES, Ecole Nationale Vétérinaire d’Alfort, Université Paris-Est, 94700 Maisons-Alfort, France; 5 Department of Veterinary Pathobiology, Center for Veterinary Health Sciences, Oklahoma State University, Stillwater, OK USA

**Keywords:** Alzheimer’s disease, Amyloid-β, Prion-like hypothesis, Amyloid-β internalization, Proteomics

## Abstract

**Background:**

Intracerebral inoculation of extracts from post-mortem human Alzheimer’s disease brains into mice produces a prion-like spreading effect of amyloid-β. The differences observed between these extracts and the synthetic peptide, in terms of amyloid-β internalization and seed and cell-to-cell transmission of cytosolic protein aggregates, suggest that brain extracts contain key contributors that enhance the prion-like effect of amyloid-β. Nevertheless, these potential partners are still unknown due to the complexity of whole brain extracts.

**Methods:**

Herein, we established a method based on sequential detergent solubilization of post-mortem samples of human brains affected by Alzheimer’s disease that strongly enrich amyloid-β aggregates by eliminating 92% of the remaining proteins. Internalization of Aβ_1–42_ from the enriched AD extracts was evaluated in vitro, and internalization of fluorescent-labeled AD extracts was also investigated in vivo. Furthermore, we carried out a molecular characterization of the Aβ-enriched fraction using label-free proteomics, studying the distribution of representative components in the amygdala and the olfactory cortex of additional human AD brain samples by immunohistochemistry.

**Results:**

Aβ_1–42_ from the enriched AD extracts are internalized into endothelial cells in vitro after 48 h. Furthermore, accumulation of fluorescent-labeled Aβ-enriched extracts into mouse microglia was observed in vivo after 4 months of intracerebral inoculation. Label-free proteomics (FDR < 0.01) characterization of the amyloid-β-enriched fraction from different post-mortem samples allowed for the identification of more than 130 proteins, several of which were significantly overrepresented (i.e., ANXA5 and HIST1H2BK; *p* < 0.05) and underrepresented (i.e., COL6A or FN1; *p* < 0.05) in the samples with Alzheimer’s disease. We were also able to identify proteins exclusively observed in Alzheimer’s disease (i.e., RNF213) or only detected in samples not affected by the disease (i.e., CNTN1) after the enrichment process. Immunohistochemistry against these proteins in additional tissues revealed their particular distribution in the amygdala and the olfactory cortex in relation to the amyloid-β plaque.

**Conclusions:**

Identification and characterization of the unique features of these extracts, in terms of amyloid-β enrichment, identification of the components, in vitro and in vivo cell internalization, and tissue distribution, constitute the best initial tool to further investigate the seeding and transmissibility proposed in the prion-like hypothesis of Alzheimer’s disease.

**Electronic supplementary material:**

The online version of this article (10.1186/s13195-019-0513-0) contains supplementary material, which is available to authorized users.

## Background

Alzheimer’s disease (AD) is the main cause of dementia worldwide [1, 2]. From a neuropathological point of view, Alzheimer’s disease (AD) is characterized by the deposition of insoluble forms of amyloid-β (Aβ) in the brain parenchyma and abnormal hyperphosphorylation of tau protein, forming, respectively, plaques and neurofibrillary tangles [3]. Even though these pathological features are well-known, the etiology of the disease is still unknown. Together with the accepted amyloid hypothesis [4, 5], the prion-like hypothesis is gaining strength to explain the etiology of neurodegenerative diseases characterized by proteinopathies—such as AD [6], whose initial stages reportedly begin in the mesial temporal structures [7]. This hypothesis is based on the capacity of Aβ to induce abnormal folding of native adjacent proteins and cell-to-cell propagation of Aβ and tau, resembling a prion-like seeding and spreading mechanism [8]. This idea is supported by the fact that inoculation of human brain extracts obtained either from patients with AD [9–11] or from APP23 mice [12] into transgenic mice [9–12] or marmosets (*Callithrix jacchus*) [13] induces the development of diffuse plaques far from the injection site [9, 10].

Prion-like spreading of AD appears to follow a three-step process. First, after injection of AD extracts, the seeds (mostly oligomers and protofibrils) are internalized by using a variety of mechanisms [14]. Second, internalized seeds nucleate the fibrillation of native monomers in the cytoplasm of the recipient cell, and a positive feedback loop starts [15]. Finally, cell-to-cell transmission of cytosolic protein aggregates starts after their release into the extracellular space in their “naked” form [15]. Different proteomic analyses have been performed using Aβ-enriched extracts [16–22] and micro-dissected plaques coupled with LC-MS/MS [23–26], in order to identify additional components which may be relevant to both transmissibility and seeding process. Usually, AD brain extracts are inoculated directly into the brain of APP23 [10, 11] or tg2576 transgenic mice [9] to evaluate the prion-like hypothesis. To our knowledge, none of the published studies has assessed the possibility of injection of fractions enriched with Aβ plaques. This study proposes a method that strongly enriches Aβ aggregates from human AD samples while retaining their ability to internalize Aβ in vitro and in vivo*.* Proteomic characterization of these extracts revealed the presence of several proteins either over- or underrepresented in AD-enriched fractions, which may contribute to plaque integrity and/or Aβ internalization.

## Materials and methods

### Human brain samples

Human tissue blocks were provided by biobanks IDIBAPS (Barcelona), BT-CIEN (Madrid), and BIOBANC-MUR (Murcia). Experimental procedures were approved by the Ethical Committee for Clinical Research of the Ciudad Real University Hospital. Twelve human brain samples containing the olfactory cortex, amygdala, and hippocampus were used (five diagnosed AD cases, six cases with no AD diagnosis, and one case with incidental plaques but no AD diagnosis; Additional file 1: Table S1). The exact locations of the brain sections used in this study are shown in Additional file 2: Figure S1.

### Enrichment of Aβ plaque-containing fractions from human brains

To obtain Aβ-enriched fractions, we used two samples from human brains with no AD diagnosis, two cases diagnosed as AD (stage VI), and one case with incidental Aβ plaques but no AD diagnosis (Additional file 1: Table S1). Approximately 10 g from each sample was cut into 1-cm^3^ blocks and homogenized in lysis buffer (20 mM Tris pH 7.4, 100 mM NaCl and 5 mM CaCl_2_) supplemented with protease inhibitor. Homogenization was performed on a mechanical homogenizer on ice. The homogenates were incubated on ice with DNase I. An efficient enrichment of Aβ plaques from the human brain was achieved using a protocol based on four centrifugation steps using Triton X (1%) and SDS (1.75%) followed by acetone precipitation (Additional file 2: Figure S2A). This protocol was applied to two human brain samples with no evidence of AD pathology (non-AD; *n* = 2), samples from two AD patients (AD; *n* = 2), and also one sample from a patient with diffuse plaques but no evidence of AD (DP; *n* = 1), for comparison. An overview of the process is shown in Additional file 2: Figure S2A. The resulting precipitate was resuspended in Hank’s solution and stored at − 20 °C.

### Determination of the efficacy of Aβ enrichment

**Fig. 1:**
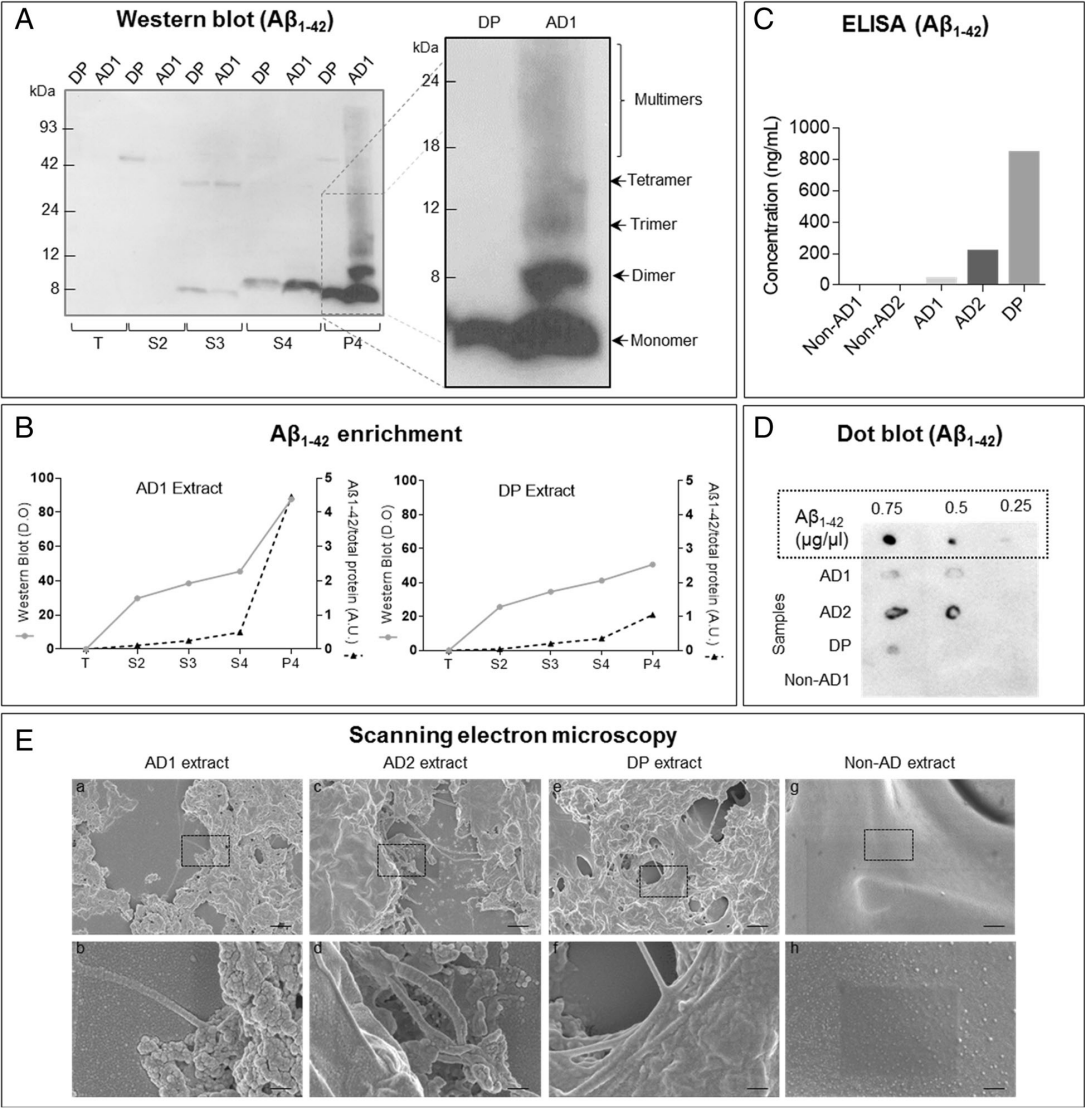
Quantification and evaluation of the Aβ-enriched extracts. **a** Western blot of AD sample 1 (AD1) and diffuse plaque sample (DP) using Aβ_1–42_ antibody. The last fraction enriched in Aβ plaques is shown boxed in order to identify Aβ monomer (4-kDa band), dimers, and multimers. **b** Ratio between western blot signal and Coomassie blue staining that reflects Aβ enrichment in the fractions. **c** ELISA of Aβ_1–42_ carried out with non-AD (non-AD1 and non-AD2), AD (AD1 and AD2), and DP samples. **c** Dot blot on the enriched extracts (P4) of the samples previously analyzed with western blot and ELISA. SEM images of AD1 extracts (a, b), AD2 extracts (c, d), DP extracts (e, f), and non-AD extracts (g, h). Scale bars: a, c, e, g, 1 μm and b, d, f, h, 200 nm

The Aβ_1–42_ content (the major component of Aβ plaques in the brain parenchyma) of the fractions was examined with dot blot (Additional file 2: Figure S2B-C) and western blot (Fig. 1A). Dot blot was carried out with 1 μl of each fraction resulting from the enrichment procedure, which was set on PVDF membranes. PVDF membranes were dried during 2 h at 60 °C and blocked with 5% BSA (VWR, Solon; OH, USA) in TTBS (200 mM Tris-HCl pH 8.8, 6 mM NaCl and 1% Tween20) for 1 h at room temperature. Membranes were blotted with Aβ_1–42_ (1:5000) and tau (1:1000) antibodies. For western blot, all the protein extracts were quantified using bicinchoninic acid assay (BCA; Sigma Aldrich). Sixty micrograms of protein for each sample (all supernatants and pellet) was prepared using 12.5-μL sample buffer (Bio-Rad), 2.5-μL reducing buffer (Bio-Rad), and deionized water, and boiled for 5 min. Samples were electrophoresed on Criterion™ XT Precast Gel 12% Bis-Tris (Bio-Rad) with XT MES running buffer (Bio-Rad) at 180 V for 45 min. The proteins were transferred to a PVDF membrane (Bio-Rad) for 50 min at 0.25 mA constant in transfer buffer (50 mM Tris-HCL, pH 8.8, 192 mM glycine, 0.02% SDS and 20% methanol). Membranes were stained with ponceau red and destained with 1% acetic acid and blocked in 5% BSA in TTBS for 1 h at room temperature and then incubated with Aβ_1–42_ antibody (1:1000; 2.5% BSA in TTBS) overnight at 4 °C. After secondary antibody incubation, the membranes were washed with TTBS and developed with Clarity Western ECL substrate according to the manufacturer’s directions. To evaluate the effectiveness of the enrichment Aβ_1–42_, western blot results were compared with Coomassie blue staining of 20 μg of all the fractions by quantifying the ratio with ImageJ.

### Quantification of Aβ_1–42_ contents in the enriched fraction

Quantification of the Aβ contents in the final enriched fraction was done by dot blot and ELISA after sonicating the samples. ELISA was performed using an Aβ42 Human ELISA Kit (Invitrogen; Vienna, Austria) following the manufacturer’s instructions. The samples were sonicated (Branson Ultrasonics™ Sonifier™ SFX150) for 1 min. Dot blot was performed as described previously using different dilutions of the extract and dilutions of the synthetic Aβ_1–42_ peptide with known concentrations.

### Scanning electron microscopy (SEM)

The presence of aggregates in the Aβ-enriched extracts was confirmed by SEM after sonication. The homogenates were diluted in methanol and centrifuged at 16100*g* for 10 min at 4 °C, twice. The resulting pellets were resuspended in pure methanol, then deposited in silicon and left to dry. Samples were metallized with gold chips and observed using a Zeiss GeminiSEM 500 scanning electron microscope.

### In vitro Aβ_1–42_ internalization into endothelial cells

Internalization of Aβ_1–42_ from enriched extracts was examined in vitro*.* As endothelial cells are the first to interact with the circulating Aβ, we used for these studies the endothelial cell line bEnd.3 (ATCC CRL-2299). Cells were cultured in DMEM containing 10% fetal bovine serum (FBS) and 1% antibiotic/antimycotic, at 37 °C and 5% CO_2_. They were then seeded in eight-well μ-Slides (Ibidi, Martinsried, Germany) and incubated with AD and non-AD extracts. After 48 h, cells were fixed and permeabilized in 4% paraformaldehyde (PFA). To analyze Aβ incorporation, cells were sequentially incubated with Aβ_1–42_ antibody (1 h) and AlexaFluor® 488-conjugated anti-mouse antibody (1 h). Cells were co-stained with 1 μg/ml Hoechst and ActinRed™ 555 and examined using an LSM 800 confocal microscope (Zeiss; Jena, Germany) with a × 63 objective.

### In vivo microinjection of Aβ-enriched fractions

In vivo experiments were carried out with female C57BL/6J mice (000654, The Jackson Laboratory, USA). Three different experimental groups were established: saline microinjection (*n* = 6), human non-AD extract microinjection (*n* = 6), and human AD Aβ-enriched extract microinjection (*n* = 6). Animals were anesthetized using vaporized isoflurane. The animals were placed in a Kopf (Tujunga, CA) stereotaxic apparatus, and the skull trepanned at the injection spot into the dorsal part of the left anterior olfactory nucleus (AONd, coordinates from Bregma [27] were AP = + 2.8 mm, *L* = 1 mm, and depth = − 2.75 mm, from the dura mater). This structure was chosen because it is preferentially vulnerable and directly involved in Aβ aggregation [28, 29]. Next, saline, 6 μg/μl of human non-AD and 6 μg/μl of human Aβ_1–42_-enriched extracts (all extracts were previously tagged with AlexaFluor® 488 nm [green] labeling dye [Molecular Probes]) were injected in a constant infusion (0.2 μl/min) for 10 min using a microsyringe (10 μL Neuros Model 1701 RN, point style 4, SYR, Hamilton Co., Nevada, USA). Animals were kept on the stereotaxic apparatus for additional 5 min to favor the diffusion of the extract before removing the syringe. The animals were housed on a standard 12/12 h light/dark cycle, at 21 °C with food and water ad libitum. All the animal research procedures described herein were in agreement with European (Directive 2010/63/EU) and Spanish (RD 53/2013) legislation on the protection of animals used for scientific purposes. All experiments described were approved by the Ethical Committee for Animal Research of the University of Castilla-La Mancha (SAF2016-75768-R).

### Isolation of mouse brain for immunostaining

Four months after injection, animals were anesthetized with a mixture of ketamine hydrochloride (1.5 mL/kg, 75 mg/kg, Ketolar, Madrid, Spain) and xylazine (0.5 mL/kg, 10 mg/kg, Xilagesic, Calier, Barcelona, Spain) and perfused with saline solution followed by 4% *w*/*v* para-formaldehyde fixative (phosphate-buffered; 0.1 M sodium phosphate, pH 7.2). Brains were post-fixed in 4% *w*/*v* paraformaldehyde, cryo-protected in 30% *w*/*v* sucrose, and coronally sectioned (50 μm) employing a freeze sliding microtome. To visualize the injection site, sections were counterstained with DAPI. To study the extracts injected into the AONd, saline, human non-AD extracts, and human Aβ_1–42_-enriched extracts were tagged with AlexaFluor® 488 nm (green) dye, following the manufacturer’s instructions. Immunofluorescence labeling against Iba-1 (1:1000) and AlexaFluor® 568 nm (1:200) was performed in order to detect microglia.

### Label-free proteomics of human brain extracts

For proteomics analysis, two AD samples, two non-AD samples, and one corresponding to a patient with pre-amyloid diffuse plaques but without evidence of AD were used (all samples were enriched as described in the previous section). The protein extracts (150 μg per sample) were concentrated on gel and analyzed by reverse phase liquid chromatography-tandem mass spectrometry (RP-LC-MS/MS) using an Easy-nLC II system coupled to a linear ion trap mass spectrometer model LTQ (Thermo Scientific) as previously described [30]. The MS/MS raw files were searched against the Uniprot–Human proteome database (70,931 entries in January 2019) (http://www.uniprot.org) using the SEQUEST algorithm (Proteome Discoverer 1.4, Thermo Scientific). The following constraints were used for the searches: tryptic cleavage after Arg and Lys, up to two missed cleavage sites, and tolerances of 1 Da for precursor ions and 0.8 Da for MS/MS fragment ions and the searches were performed allowing optional Met oxidation and Cys carbamidomethylation. A false discovery rate (FDR < 0.01) and at least two peptides per protein was considered as a condition for successful peptide assignments. For the semiquantitative analysis of proteins, the total number of peptide-spectrum matches (PSMs) for each protein was normalized against the total number of PSMs in each sample and compared between AD and non-AD samples using the chi-square test (*p* < 0.05).

Gene ontology analysis study was carried out with the proteomic profiles obtained for both AD and non-AD-enriched extracts to identify overrepresentation profiles. To that end, we use GOrilla (http://cbl-gorilla.cs.technion.ac.il; [31]), a bioinformatics tool previously used in several studies (i.e., [32]). As a background, we used the most recent database of a global quantitative analysis of the human brain proteome in Alzheimer’s disease, extracted from [33] (16,559 recognized proteins in GOrilla; database updated June 2019). We set *p* value to 10^− 6^ in order to avoid unreliable data. Gene ontology was investigated at three levels: biological process, biological function, and biological component.

### Immunofluorescence and immunohistochemical procedures

Immunofluorescence analysis was performed in 4% phosphate-buffered formaldehyde-fixed samples. Afterwards, all blocks were post-fixed in fresh 4% phosphate-buffered paraformaldehyde for 45 days. Coronal sections of the amygdala, olfactory cortex, and hippocampus (50 μm) were obtained using a freezing sliding microtome Microm HM 450. For these experiments, a total of six antibodies against Aβ_1–42_, RNF213, COL6A, ANXA5, CNTN1, and GFAP were used (Additional file 1: Table S2). Tissue antigenicity was unmasked by boiling the tissue under pressure for 2 min in citrate buffer. Sections were immersed in formic acid for 3 min and rinsed in phosphate buffer. Endogenous peroxidase activity was inhibited by a 30-min bath in 1% H_2_O_2_ in phosphate-buffered saline. Blocking consisted of 5% NDS+ 0.3% Triton X-100 in PBS. Sections were incubated overnight at 4 °C with primary antibodies containing 0.3% Triton X-100 and 5% normal serum in phosphate-buffered saline. Controls included omitting primary or secondary antibodies. Sections were counterstained using DAPI (Santa Cruz Biotechnology; Inc.; Sc-3598) or Nissl and coverslipped with PVA-DABCO or DPX after dehydrating. Human tissue autofluorescence was not reduced. Images were captured using an LSM 800 confocal microscope and analyzed using ZEN software.

## Results

### Detergent-centrifugation method allows efficient enrichment of insoluble Aβ_1–42_

The presence of Aβ_1–42_ in the five samples during the different steps was monitored by dot blot [Additional file 2: Figure S2B (non-AD1, AD1 and DP) and Additional file 2: Figure S2C (non-AD2 and AD2)]. As expected, the results showed no Aβ_1–42_ in the non-AD samples. The SDS 1.75% supernatants (S3–S4) showed immunostaining both in AD and DP samples, suggesting that part of the soluble Aβ_1–42_ was diluted using this concentration of SDS. In AD samples, the pellet from the last step was extremely difficult to reconstitute and showed a strong Aβ_1–42_ signal (probably corresponding to the Aβ plaques). Interestingly, the DP samples did not show reactivity in the insoluble fraction (Additional file 2: Figure S2B), and tau protein was mainly eliminated in the second wash with SDS (Additional file 2: Figure S2D). A 4-kDa band was observed with strong immunoreactivity against Aβ_1–42_ in the last fraction of the AD and DP samples (Fig. 1A), probably corresponding to monomeric Aβ_1–42_. Aβ_1–42_ dimers, trimers, and oligomers appeared only in the AD samples (Fig. 1A, right), indicating that AD and DP experience different aggregation strengths.

Estimation of Aβ_1–42_ enrichment (Fig. 1B) was attempted by comparing western blot and Coomassie blue staining (Additional file 2: Figure S2E). In AD samples, the concentration of Aβ_1–42_ increased according to the ratio western blot/Coomassie blue staining (Fig. 1B, left). In the DP sample, the ratio did not change (Fig. 1B, right). In order to accurately quantify Aβ_1–42_ concentration, a human Aβ_1–42_ ELISA (Invitrogen; Fig. 1C) was performed. Despite the sensitivity of the kit, non-AD samples did not show immunoreactivity against Aβ_1–42_. It is especially relevant that although AD samples contained 50 ng/mL (AD1) and 210 ng/mL (AD2) of Aβ_1–42_, the DP sample showed a higher concentration of Aβ_1–42_ (over 0.8 μg/ml). This result is completely opposite to that obtained with western blot and dot blot. To quantify Aβ concentration in the extracts, dot blot with diluted concentrations of the Aβ-enriched fractions and known dilutions of synthetic Aβ_1–42_ [34] (Sigma Aldrich; Fig. 1D) was carried out. The results indicated a concentration of Aβ_1–42_ in the sample ranging from 0.1 to 0.5 μg/μL. Although we are aware that neither of the methods developed so far for insoluble Aβ_1–42_ quantification are accurate, the examination of our samples by electron microscopy revealed the presence of aggregates (with the presence of fibrils) in AD (Fig. 1E, a–d) and DP (Fig. 1E, e–f) sonicated extracts, which were not observed in non-AD samples (Fig. 1E, g–h).

### Aβ internalization from the enriched fractions into cultured microvascular endothelial cells

**Fig. 2:**
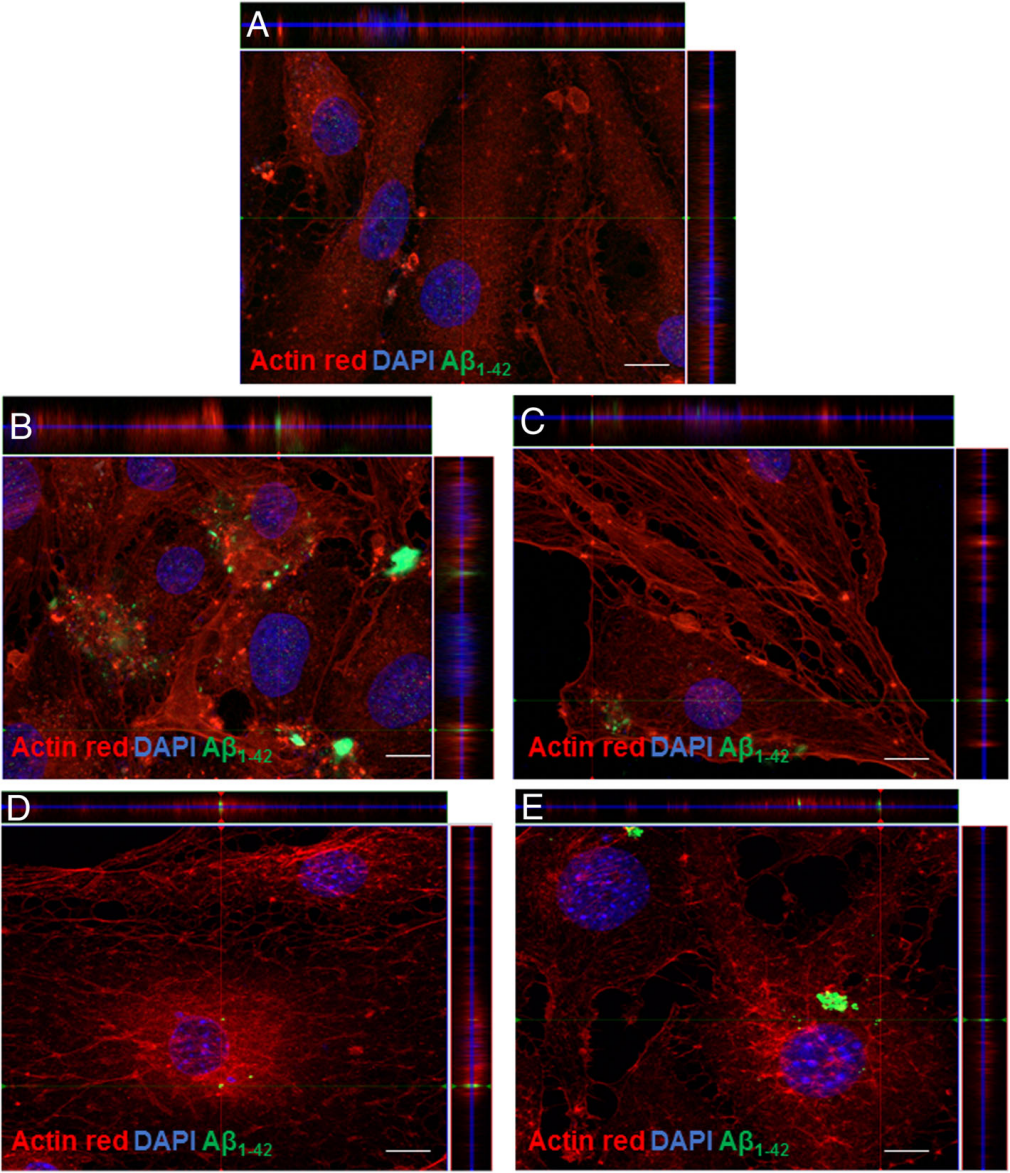
Immunofluorescence against Aβ_1–42_ in ActinRed™ 555-labeled endothelial cells (bEnd.3) after 48-h incubation with the enriched extracts. **a** Non-AD extracts. **b**, **c** Two representative cells after incubation with AD1-enriched extracts. **d**, **e** Two representative cells after incubation with AD2-enriched extracts. Z-stacks are shown on the top and right sides of each image. Pictures were acquired using a confocal microscope with a × 63 objective. Scale bars **a**–**d** = 10 μm


Our Aβ plaque enrichment protocol was aimed at maintaining the plaque integrity and to preserve its prion-like properties. Therefore, we first evaluated the in vitro incorporation Aβ_1–42_ internalization into bEnd.3 cells were tested by adding AD-enriched extracts (a volume corresponding to approximately 1 μg of Aβ_1–42_ according to dot blot quantification) to the cell culture. Figure 2 shows immunofluorescence against the Aβ_1–42_ antibody and ActinRed™ 555 labeling after 48-h incubation with non-AD extracts (Fig. 2a) and Aβ-enriched extracts from AD1 (Fig. 2b, c) and AD2 (Fig. 2d, e). This strategy allowed us to visualize internalized Aβ_1–42_ in vesicle-like structures using confocal z-stacks (Fig. 2b–e)*.* **Additional file 3: Video S1.** shows different angles of the Aβ_1–42_ internalized in the cell from Fig. 2b*.*

Additional file 3: Video S1 (MP4 2166 kb)


### In vivo injection of Aβ plaque-enriched extracts in mouse brain and Aβ_1–42_ internalization

**Fig. 3:**
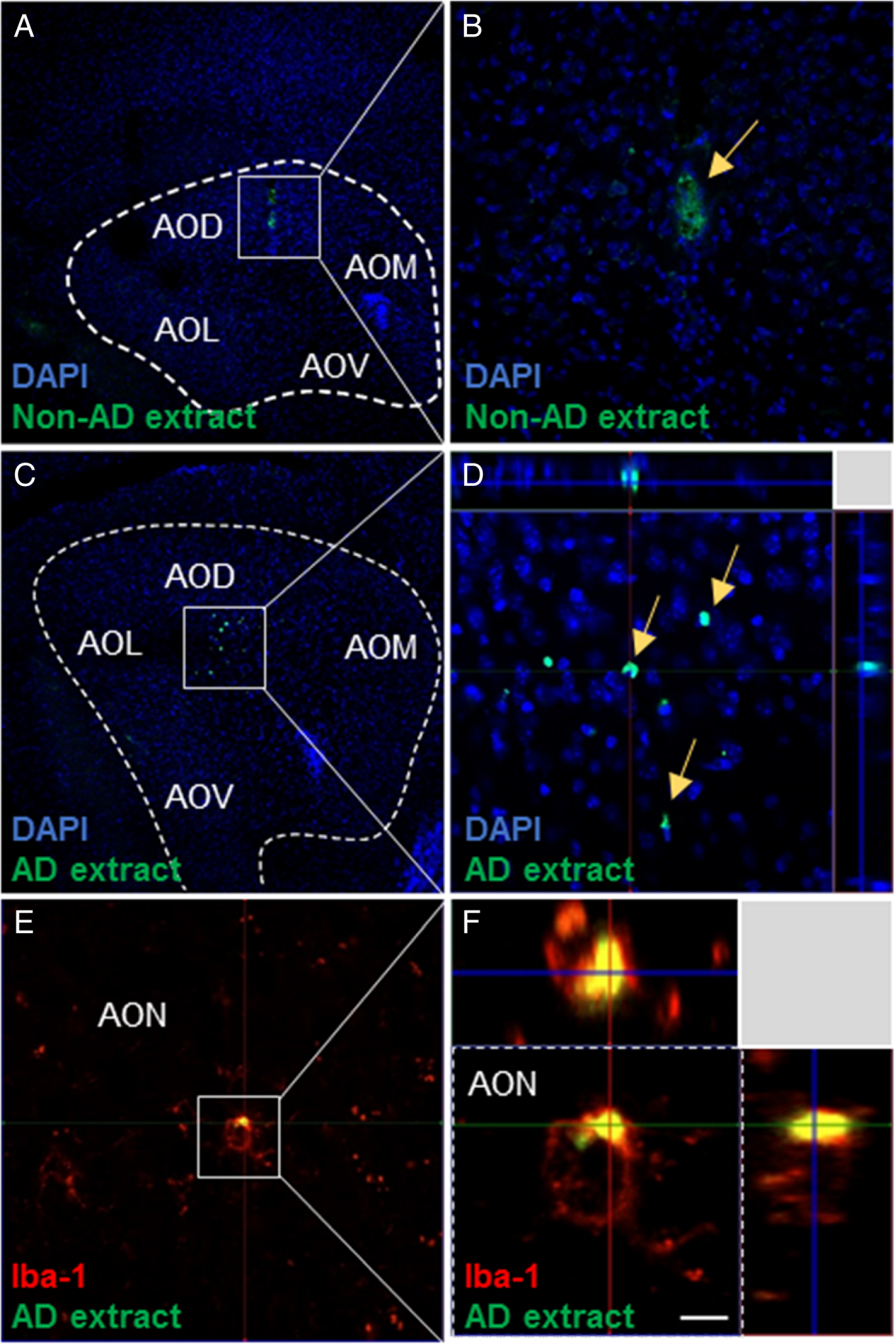
Microinjection and internalization of Aβ_1–42_ in the dorsal anterior olfactory nucleus. Images show the region where the AlexaFluor 488-labeled extracts were injected. Human non-AD extracts (**a**, **b**) and human Aβ_1–42_-enriched extracts (**c**, **d**). Nuclei are shown in blue (DAPI), and labeled extracts appear in green. Images e and f correspond to the tissue of samples injected with labeled extracts (green) together with Iba-1 labeling (red). Z-stack images are also shown. Scale bars **a**, **c** = 130 μm, **b** = 20 μm, **d** = 40 μm, **e** = 10 μm, **f** = 3 μm. AO anterior olfatory nucleus (D, dorsal; L, lateral; M, medial; V, ventral)

Aβ-enriched extracts tagged with AlexaFluor® 488 nm (green) were injected into the dorsal part of the anterior olfactory nucleus. Labeled saline injections (images not shown) did not reveal any fluorescence near the injection site. Inoculation with the extracts obtained non-AD sample allowed for the identification of the injection site, but no fluorescence was detected inside the cells (Fig. 3a, b). In contrast, the fluorescence of the sections 4 months after inoculation of Aβ_1–42_-containing extracts could be observed inside the cells closer to the injection location (Fig. 3c, d). Internalization of human Aβ extracts (green) in microglia was also examined using Iba-1 antibody (red; Fig. 3e). Figure 3f shows an Iba-1-positive cell within the z-stack.

### Proteomic characterization of Aβ-enriched extracts

Next, the enriched fraction containing insoluble Aβ was characterized in order to identify possible protein components bound to the Aβ plaque that had not been observed in the non-AD extracts. To that end, proteomic analysis on enriched extracts of two AD and two non-AD samples was performed. Specifically, 149 and 133 proteins were identified in AD-enriched samples, and 144 and 131 in non-AD samples (Additional file 4: Table S3.) In AD samples, six of these proteins were overrepresented and three proteins were underrepresented (Table 1). Proteins that were exclusively detected in AD or non-AD extracts were also examined (Table 2; 1% FDR, target-decoy database approach). A total of 34 proteins were identified at least in one of the two AD samples, while 12 proteins were identified in the non-AD extracts, which probably correspond to proteins that decrease in AD-enriched extracts. Several proteins previously related to AD were identified as overrepresented in the Aβ-enriched fraction [APOE [35], ANXA2 [36], or MAPT [37]]. The rest of the identified proteins are shown in Table 2.

**Table 1:** Proteins overrepresented in Aβ-enriched fraction and proteins overrepresented in non-AD samples. The MS/MS raw files were examined against the universal protein database UniProt (70,931 entries in January 2019) (http://www.uniprot.org), using the SEQUEST algorithm (Proteome Discoverer 1.4, Thermo Fisher Scientific). Fold of change; *P* value < 0.05. *PSM* peptide-spectrum matches, *x̄* average, *ND-AD* not detected in Alzheimer extracts, *ND-non-AD* not detected in control extracts

Symbol	Entrez gene name	ID	PSM non-AD1	PSM non-AD2	PSM AD1	PSM AD2	x̄ non-AD	x̄ AD	Fold of change	*p* value
A) Proteins overrepresented in AD enriched fractions										
GFAP	Glial fibrillary acidic protein	P14136	36	48	71	100	42	85.5	2.04	1E−4
MBP	Myelin basic protein	J3QL64	11	14	41	17	12.5	29	2.32	0.013
SH2D3C	SH2 domain-containing protein 3C (fragment)	Q5JU32	19	2	47	2	10.5	24.5	2.33	0.019
ANXA5	Annexin A5	P08758	0	0	4	15	0	9.5	ND–non AD	0.001
HIST1H2BK	Histone H2B type 1-K	O60814	0	0	9	1	0	5	ND–non AD	0.025
ANXA2	Annexin A2	H0YMM1	0	0	2	6	0	4	ND–non AD	0.045
B) Proteins overrepresented in non-AD enriched fractions										
COL6A3	Collagen alpha-3(VI) chain	E7ENL6	13	28	0	7	20.5	3.5	5.86	6E−4
FGB	Fibrinogen beta chain	P02675	15	23	0	15	19	7.5	2.53	0.034
FN1	Fibronectin	P02751	2	5	0	0	3.5	0	ND–AD	0.045

**Table 2:** Proteins found exclusively in AD extracts and proteins found exclusively in non-AD extracts

Symbol	Entrez gene name	ID	PSM
A) Proteins found exclusively in AD extracts			
ANXA5	Annexin A5	P08758	19
HIST1H2BK	Histone cluster 1 H2B family member k	O60814	10
ANXA2	Annexin A2	H0YMM1	8
DNAH11	Dynein axonemal heavy chain 11	Q96DT5	5
HIST1H2BA	Histone cluster 1 H2B family member a	Q96A08	5
XRCC5	X-ray repair cross complementing 5	P13010	5
APOE	Apolipoprotein E	P02649	4
CFAP43	Cilia and flagella associated protein 43	Q8NDM7	4
RNF213	Ring finger protein 213	A0A0A0MTR7	4
RPL4	Ribosomal protein L4	H3BM89	4
HBB	Hemoglobin subunit beta	F8W6P5	3
MAPT	Microtubule associated protein tau	I3L170	3
NME1-NME2	NME1-NME2 readthrough	J3KPD9	3
PIK3R2	Phosphoinositide-3-kinase regulatory subunit 2	E9PFP1	3
SEPT2	Septin 2	B5MCX3	3
SYNE2	Spectrin repeat containing nuclear envelope protein 2	G3V5X4	3
CCDC171	Coiled-coil domain-containing 171	H0Y5M5	2
CD9	CD9 molecule	A6NNI4	2
CFAP47	Cilia and flagella associated protein 47	A0A140T8X2	2
DHH	Desert hedgehog	O43323	2
EFHB	Ef-hand domain family member B	Q8N7U6	2
ESRP1	Epithelial splicing regulatory protein 1	Q6NXG1	2
IQSEC2	IQ motif and sec7 domain 2	Q5JU85	2
LRP1B	LDL receptor-related protein 1B	Q9NZR2	2
MAG	Myelin-associated glycoprotein	M0QZU4	2
MARCH1	Membrane-associated ring-ch-type finger 1	D6RGC4	2
MYBPC3	Myosin binding protein C, cardiac	A0A0A0MQU5	2
NME1	NME/NM23 nucleoside diphosphate kinase 1	P15531	2
ODF2	Outer dense fiber of sperm tails 2	Q5BJF6	2
PLPPR4	Phospholipid phosphatase related 4	Q7Z2D5	2
PLXNC1	Plexin C1	O60486	2
SCUBE1	Signal peptide, CUB domain and EGF like domain-containing 1	A0A087X285	2
SLC12A6	Solute carrier family 12 member 6	B3KXX3	2
TGFBI	Transforming growth factor beta induced	H0Y8L3	2
B) Proteins found exclusively in non-AD extracts			
FN1	Fibronectin 1	P02751	7
LAMA5	Laminin subunit alpha 5	O15230	5
ATP5F1B	ATP synthase F1 subunit beta	P06576	4
DNAH12	Dynein axonemal heavy chain 12	Q6ZR08	3
ACTN1	Actinin alpha 1	H0YJ11	2
ARHGEF26	Rho guanine nucleotide exchange factor 26	Q96DR7	2
CEP152	Centrosomal protein 152	O94986	2
CFL1	Cofilin 1	E9PLJ3	2
CNTN1	Contactin 1	Q12860	2
CWC25	CWC25 spliceosome associated protein homolog	Q9NXE8	2
DIO3	Iodothyronine deiodinase 3	P55073	2
GDI1	GDP dissociation inhibitor 1	P31150	2

GO enrichment analysis was carried out with the proteins observed in the enriched samples of both AD and non-AD, using whole brain proteome as background. This study retrieved significant results when *biological process* was inquired (Additional file 2: Figure S3). Thus, negative regulation of wound healing and homeostasis were identified an AD while cell morphogenesis and cell junction assembly appeared in the non-AD samples.

### Distribution of the proteins in the amygdala and olfactory cortex sections

**Fig. 4:**
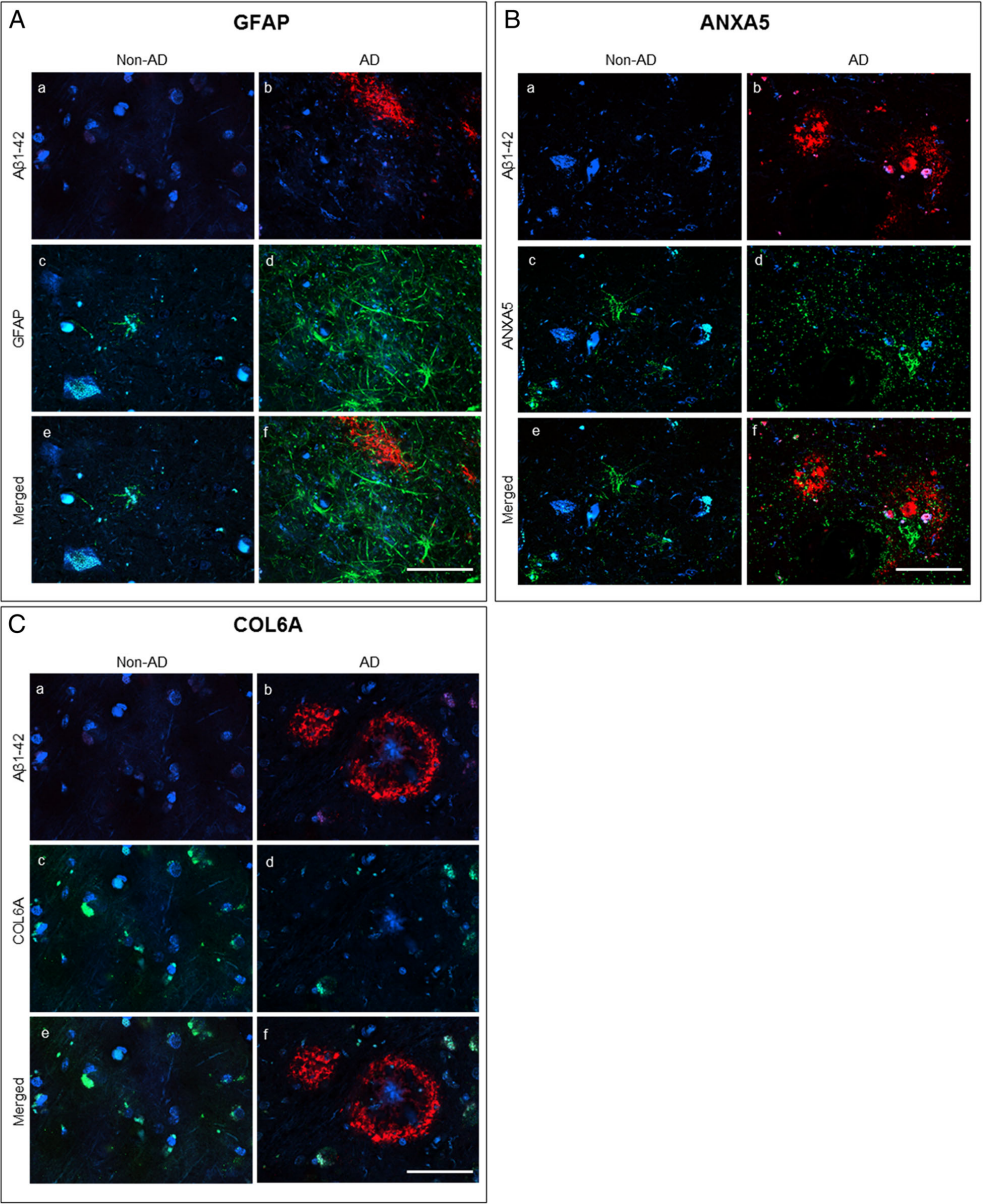
Double immunofluorescence against Aβ_1–42,_ and GFAP (**A**), Aβ_1–42_ and ANXA5 (**B**) and Aβ_1–42_ and COL6A (**C**). Confocal images of amygdala sections of human AD samples and non-AD samples, used to study the distribution of GFAP (green, **A**), ANXA5 (green, **B**), or COL6A (green, **C**). Immunostaining against Aβ_1–42_ (red, a–b) was also included to identify the Aβ plaques. Nuclei are labeled in blue with DAPI. Calibration bars 50 μm

GFAP, ANXA5, and COL6A expression was evaluated by immunofluorescence. Since proteomics had been performed in regions containing mostly amygdala (see Additional file 2: Figure S1), immunohistochemical experiments were performed in the same regions using additional AD samples. Furthermore, several of the proteins in the olfactory cortex area were also examined in order to evaluate whether the changes induced in AD could also be observed in this section of the brain—since olfactory deficits usually precede the clinical onset of cognitive and memory deficits in AD pathology [38]. GFAP expression was examined as a control of the enrichment process, since it has been previously shown to co-localize with the Aβ plaque in AD [39]. The results indicated that although there was co-localization between Aβ_1–42_ and GFAP, GFAP immunolabeling in AD could be observed in all the tissue (Fig. 4A). ANXA5 was also overrepresented in Aβ_1–42_ extracts. Immunostaining was higher in AD samples than in non-AD samples in both the amygdala (Fig. 4B) and the olfactory cortex (Additional file 2: Figure S4A). However, in contrast with GFAP, the ANXA5 signal increase was especially noticeable surrounding the plaques in human AD brain samples. As for COL6A, which was highly represented in non-AD samples, it also showed reduced immunostaining in AD samples (Fig. 4C).

**Fig. 5:**
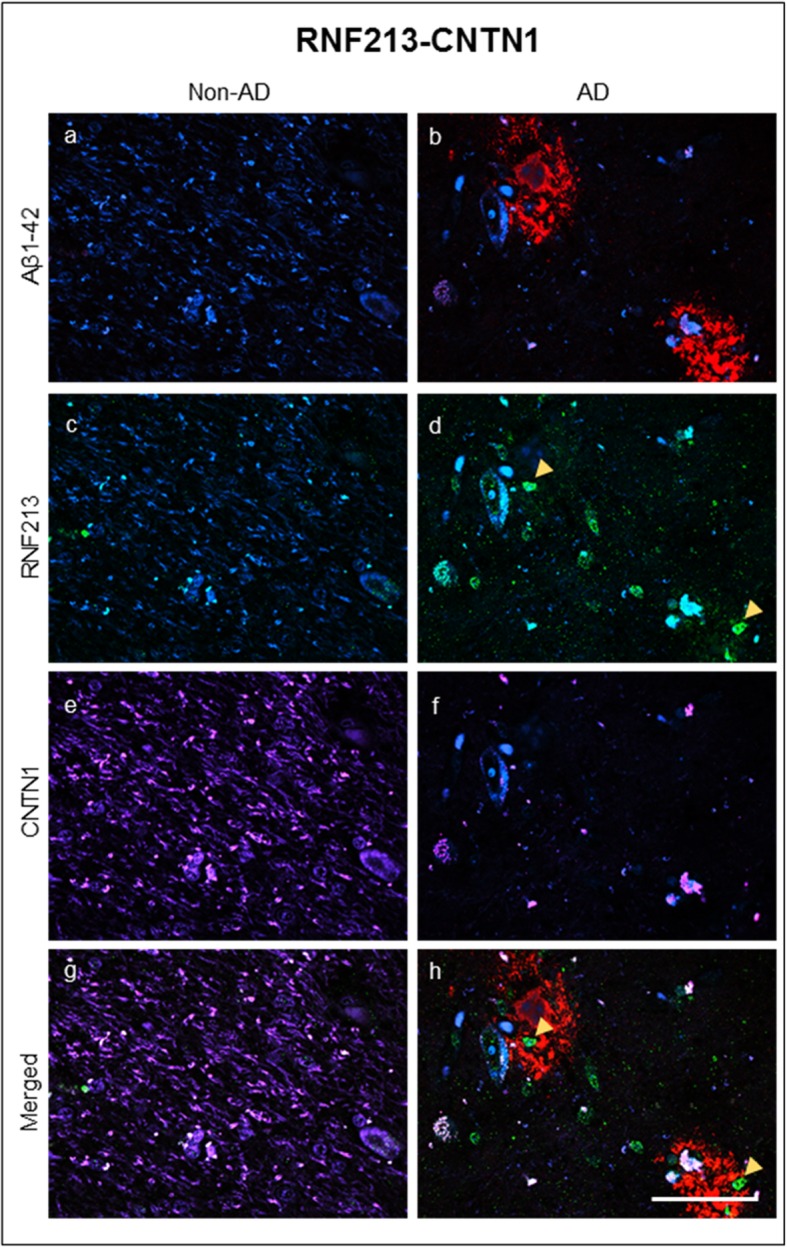
Triple immunofluorescence against Aβ_1–42,_ RNF213, and CNTN1. Confocal images showing a triple-immunofluorescence against Aβ_1–42_ (**a**, **b**), RNF213 (**c**, **d**), and CNTN1 (**e**, **f**) in coronal sections of non-AD (**a**, **c**, **e**, and **g**) and AD human brain (**b**, **d**, **f**, and **h**). Calibration bar **a**–**h** 50 μm. Yellow arrowheads indicate the highly RNF213 immunofluorescence identified nuclei

Finally, a triple immunofluorescence was carried out in amygdala with Aβ_1–42_ (Fig. 5a, b, red) and two of the proteins observed exclusively in AD samples (RNF213; Fig. 5c, d, green) and non-AD samples (CNTN1; Fig. 5e, f, purple). It is especially relevant that CNTN1 expression in non-AD samples was strong and widespread when compared to AD samples (Fig. 5e, f). On the other hand, RNF213 immunostaining (which, as expected, appears in the nuclei of the cells) seems to be overexpressed in AD samples. Together with the Aβ_1–42_ fluorescence (Fig. 5g, h), a noteworthy increase in RNF213 intensity (twofold increase) could be appreciated when the nuclei were located into Aβ plaques (arrow). Similar experiments performed in the olfactory cortex of AD patients revealed that CNTN1 was also highly abundant in non-AD samples in this section of the brain (Additional file 2: Figure S4B*)* while RNF213 appeared strongly associated within the limits of the Aβ plaque (Additional file 2: Figure S4B).

## Discussion

### Aβ enrichment from AD patients and pre-amyloid diffuse plaques

Several laboratories have developed protocols to extract Aβ and other components from AD plaques [17, 19, 22, 40] although those with higher success rates (70–99% formic acid; [22]) involve aggressive treatments of the Aβ plaque and solubilize the core structure of amyloid fibrils [22]. In this study, we aimed at preserving as much fibrillar structures as possible and therefore we used a four-step enrichment protocol based on detergent centrifugations. We avoided the combination of detergent extraction with high molarity urea or guanidinium salts as it may result in increased solubilization of fibrillar structures [41]. We also avoided sonication during the process as it has been shown that it produces the redistribution of Aβ species from plaques to more soluble fractions [41].

In AD, Aβ aggregates start as pre-amyloid diffuse plaques [42] that contain smaller amounts of Aβ and are not as organized into fibrils as in more advanced stages. In this study, a strong precipitate was observed in Aβ-enriched samples of stage VI AD patients, and dimers and oligomers remained in western blot experiments. The presence of such insoluble material was not observed in non-AD samples and barely observed in the extract from the patient with diffuse plaques. Nevertheless, in order to obtain accurate information of the soluble Aβ that was present in each enriched extract used for the different experiments, we avoided the use of formic acid or guanidinium salts for ELISA. Quantification in such conditions uncovered the presence of five to ten times higher Aβ_1–42_ in DP than in AD. In this sense, it has been shown that ELISA usually works fine with soluble Aβ but not with insoluble Aβ aggregates as it underestimates the concentration of total Aβ_1–42_ of the Aβ plaque [43]. Taking into consideration the solubility of Aβ in the AD samples, it was not possible to ascertain their Aβ_1–42_ concentration, and only dot blot analysis (which has previously been used for this purpose) [34] gave an approximated concentration of 0.5 μg/μl of Aβ in the final pellet. Although we are aware that the presence of insoluble material may introduce some bias in the process of protein quantification, as far as we know, this concentration constitutes the highest Aβ enrichment tested to date in vivo and in vitro.

### Labeled extracts from Aβ-enriched fractions are internalized in vitro and in vivo

This study provides the first evidence that AD-enriched extracts, but not non-AD extracts, are efficiently incorporated into the cells surrounding the injection site 4 months post-injection, which constitutes the first step toward Aβ transmissibility. Additional labeling with Iba-1 demonstrates that among the cells that incorporate labeled extracts of AD patients in vivo there are glial cells (microglia). This fact has been previously described in vitro [34], where it was proposed that Aβ_1–42_ protofibrils were more efficiently internalized by microglia than monomers [34]. In fact, microglia might play a crucial role in AD [44]. We cannot assure that the long-lived component of the extracts incorporated into Iba-1-positive cells is exclusively Aβ_1–42_ as we labeled whole Aβ_1–42_-enriched extracts. In fact, there exists the possibility that the observed fluorescence corresponds to additional labeled proteins that are not enriched in control extracts, as these extracts are not internalized. In this sense, previous experiments injecting preparations of soluble or fibrillar synthetic Aβ_40_, Aβ_42_, or a mixture of both [11] and Aβ_1–40_, Aβ_1–42_, and Aβ_40–1_ synthetic Aβ peptides [13] did not generate such a prion-like effect as AD brain extracts, suggesting that these extracts may contain unique features important for the internalization and transmission of the seeding. Also, further attempts to initiate the aggregation of Aβ in vivo with synthetic peptides in combination with several of the components known to be associated with the Aβ plaque, such as ApoE [11], did not reach success reinforcing the idea that other plaque components should be participating in this phenomenon. This is especially relevant, and we are currently undergoing further experiments to identify the nature of the incorporated proteins. In addition, our study also found that cultured endothelial cells, as the primary components of the blood–brain barrier, uptake Aβ extracts from Aβ-enriched fractions in what may constitute the pathway for Aβ to reach the bloodstream.

### Proteomics analysis identified components that were over- and underrepresented in plaque-containing AD extracts

Previous studies [39, 45] have related a hyper-reactivity of the GFAP protein to Aβ deposition. In this study, the GFAP signal was found to be intense in the amygdala of AD patients, corroborating those findings. Two members of the annexin family were also enriched in AD extracts. One of them, annexin A5 (ANXA5), has been proposed as an AD biomarker since its plasma levels, commonly used to detect apoptotic cells, are significantly higher in AD patients [46, 47]. Intense ANXA5 immunoreactive spots were observed in an AD transgenic mouse model [47]. Our study shows for the first time that the ANXA5 signal is especially intense several micrometers around the plaque. In this sense, GO enrichment analysis revealed overrepresentation of two biological processes in AD extracts, where ANXA5, together with ANXA2, APOE, and CD9 proteins take part: negative regulation of wound healing and homeostasis. These biological processes are not found in non-AD extracts and enhance the potential importance of these four proteins for systemic inflammation, or other processes that lead to the brain homeostasis collapse in advanced Alzheimer’s disease stages [48].

It is especially intriguing that the presence of HIST1H2BK among the most significant proteins is overrepresented in Aβ-enriched extracts. This fact has been observed for histone H3 previously in full extracts off AD by iTRAQ [49] and in laser proteomics of the plaques for histone H4 [25]. Search for ligands of b-APP using ligand blotting showed strong affinity for histones specially H4 [50], and although we do not know whether histones bind or not to APP-derived peptides, their consistent finding in proteomic studies points toward a deep histone-amyloid plaque relation. This fact is especially relevant and deserves a deeper analysis, even more if we take into account that extracellular histones induce inflammation and other toxic effects [51, 52]. It is also relevant the result that suggests that fibronectin is reduced in AD extracts vs non-AD as major changes in the molecular composition of the vascular basement membranes (BM) are observed in acute and chronic neuropathological settings [53]. Other proteins of the extracellular matrix (ECM) intimately related with the BM such as fibrinogen or laminin A5 are more abundant in non-AD extracts while no differences were found on tubulins or vimentin between non-AD and AD samples. Therefore, the dissimilar distribution of these structural components of the BM and the ECM between AD and non-AD extracts reflects alteration of the BM that occurs in AD [54].

It also shows that the alpha-3 subunit of type VI collagen decreases in patients with AD. Collagen VI is an extracellular matrix protein which, according to previous studies [55], protects neurons from the toxicity of Aβ peptides. Consequently, brains with collagen VI deficiency can be expected to show greater vulnerability to apoptosis, due to alterations in oxidative stress triggered by Aβ deposits [56]. This protein could be a promising focus for future treatments.

On the other hand, one of the most interesting proteins observed exclusively in both samples of Aβ-enriched extracts is the E3 ubiquitin-protein ligase RNF213 [57]. RNA expression of the gene encoding this protein was found in genomic microarrays from AD patients [57]. Recent studies suggest that RNF213 plays an important role in cerebral blood flow maintenance under ischemic conditions by affecting angiogenesis [58], processes whose alteration usually precede development of amyloid plaques in AD patients and AD animal models [59]. Immunoreactivity is mostly observed in the nucleus (increased in those located inside the plaque), although, in the olfactory cortex, the protein is also found within the plaque. The distribution of RNF213 immunostaining reveals a potential direct Aβ plaque biomarker. Contrary to RNF213, CNTN1 was highly expressed in non-AD, as opposed to AD, samples. The contactin family has been known to interact with the extracellular domain of amyloid precursor protein (APP). For instance, contactin-2 regulates APP cleavage by secretases, a process important for neurotoxic Aβ liberation and production of the APP intracellular domain [60]. Also, it is implied in synaptic plasticity [61].

It is interesting that a number of specific calcium binding (ANXA2, ANXA5, and FN1; [62–64]) or calcium-sensitive (COL6A3 [65]) proteins are differentially enriched in proteomics of both AD and non-AD extracts. This fact may be related to the importance that calcium plays in the progression of AD ([66]) where calcium increases are associated with increased production of Aβ and also with reductions in macroautophagy—a clearance pathway for intracellular aggregates—[67].

## Conclusions

In conclusion, the obtained enriched Aβ fractions of human AD brains retain their ability to be internalized in vitro by cultured cells, but also the intracellular detection in vivo of the labeled extracts was marked after 4 months post-inoculation. Therefore, the enriched Aβ extract allows for easy traceability by immunofluorescence as the increased concentration of Aβ plaques amplifies the cell exposure to Aβ. We have deeply characterized the components of Aβ-enriched fractions identifying several proteins with dissimilar distribution between AD and non-AD extracts and investigating the distribution of the most representative findings in amygdala and olfactory cortex. These unique characteristics in terms of Aβ enrichment and extract characterization made these extracts the finest tool to further investigate seeding and transmissibility of the proposed prion-like hypothesis of AD.

## Additional files

Additional file 1: **Table S1.** Data of patients used in the present study. AD, Alzheimer disease; non-AD, non-Alzheimer disease; M, Male; F, Female; y, years; P, Proteomic study; E, ELISA; D, dot blot; I, immunofluorescence. *Human brain tissue used for the optimization of the enrichment protocol. Table S2. Antibodies used in the present study. (PPTX 47 kb)

Additional file 2: **Figure S1.** Schemes showing the medial view of the brain and the corresponding levels: A) Coronal sections of olfactory (B) and amygdaloid (C) areas analyzed [Bregma − 5.8 and − 6.7 mm respectively, according to [68]]. The corresponding tissue blocks (D, E) and Nissl-stained, mosaic-reconstructed sections (F, G) are also illustrated. Calibration bars 1 cm. A: amygdala; aic: anterior limb of internal capsule; AONc: anterior olfactory nucleus, cortical part; C: claustrum; cc: corpus callosum; Cd: caudate nucleus; HiH: hippocampal head; ic: internal capsule; LV: lateral ventricle; OlfA: olfactory area; Pir: piriform cortex; PHG: parahippocampal gyrus; Pu: putamen; SG: straight gyrus; TLV: temporal horn of lateral ventricle; un: uncus; Ent: Entorhinal cortex; PRC: perirhinal cortex. **Figure S2.** Overview of the protocol for the enrichment of Aβ plaques (A). Dot blot of the steps of Aβ plaque enrichment using Aβ_1–42_ antibody (B–C) and tau antibody (D). Coomassie blue of the steps of Aβ enrichment. AD, Alzheimer disease; Non-AD, non-Alzheimer disease; DP, diffuse plaques; LB, lysis buffer; T, total extract; S, supernatant; P, pellet. Numbers indicate steps in the procedure. **Figure S3.** Gene ontology overrepresentation study. “*p* value” is the enrichment *p* value computed according to the mHG or HG model. “FDR q value” is the correction of the above *p* value for multiple testing using the Benjamini and Hochberg (1995) method. **Figure S4.** Double immunofluorescence against Aβ_1–42_, and ANXA5 (A), and triple immunofluorescence against Aβ_1–42_, RNF213 and CNTN1 (B). Confocal images of human olfactory cortex sections of human AD samples and non-AD samples to study the distribution of ANXA5 (green, A), RNF213 (green, B), or CNTN1 (purple, B). Immunostaining against Aβ_1–42_ (red, A and B) was also included to identify Aβ plaques. Nuclei are labeled in blue with DAPI. The arrow indicates nuclei located inside the plaque. Calibration bars 50 μm. (PPTX 11881 kb)

Additional file 4: **Table S3.** Proteins identified by reverse-phase liquid chromatography coupled to tandem mass spectrometry (RP-LC-MS/MS) in AD1, AD2, non-AD1, and non-AD2 extracts. PSM, peptide-spectrum matches. Coverage: % of the protein identified. #peptides, number of the identified peptides corresponding to the identified protein. (XLSX 60 kb)

## Data Availability

The data generated during in this study is included in this article and its additional files.

## Abbreviations

AD: Alzheimer’s disease
ANXA2: Annexin A2
ANXA5: Annexin A5
AONd: Dorsal part of the anterior olfactory nucleus
APOE: Apolipoprotein E
APP: Amyloid precursor protein
Aβ: Amyloid beta
BCA: Bicinchoninic acid assay
BM: Basement membranes
BSA: Bovine serum albumin
Cc: Corpus callosum
Cd: Caudate nucleus
CNTN1: Contactin 1
COL6A: Collagen alpha-3(VI) chain
DMEM: Dulbecco’s modified Eagle medium
DPX: Dstyrene plasticizer xylene
ECM: Extracellular matrix
ELISA: Enzyme-linked immunosorbent assay
Ent: Entorhinal cortex
FBS: Fetal bovine serum
FDR: False discovery rate
GFAP: Glial fibrillary acidic protein
HiH: Hippocampal head
HIST1H2BK: Histone cluster 1 H2B family member k
ic: Internal capsule
iTRAQ: Isobaric tags for relative and absolute quantitation
LC-MS/MS: Liquid chromatography-tandem mass spectrometry
LV: Lateral ventricle
MAPT: Microtubule-associated protein tau
NDS: Normal donkey serum
OlfA: Olfactory area
PBS: Phosphate-buffered saline
PFA: Paraformaldehyde
PHG: Parahippocampal gyrus
Pir: Piriform cortex
PRC: Perirhinal cortex
PSM: Peptide-spectrum matches
Pu: Putamen
PVA-DABCO: Polyvinylalcohol-1,4 diazoabicyclo [2.2.2] octane
RNF213: Ring finger protein 213
RPLC-MS/MS: Reverse phase liquid chromatography-tandem mass spectrometry
SDS: Sodium dodecyl sulfate
SEM: Scanning electron microscopy
SG: Straight gyrus
TLV: Temporal horn of the lateral ventricle
Un: Uncus
