# Supplementary material for: Human amyloid-β enriched extracts: evaluation of in vitro and in vivo internalization and molecular characterization
Source: Alzheimers Res Ther. 2019 Jun 29;11:56. doi: 10.1186/s13195-019-0513-0 (PMC6599264; doi:10.1186/s13195-019-0513-0)
Supplement: Supplementary file 2 — Table S1. Data of patients used in the present study. AD, Alzheimer disease; non-AD, non-Alzheimer disease; M, Male; F, Female; y, years; P, Proteomic study; E, ELISA; D, dot blot; I, immunofluorescence. *Human brain tissue used for the optimization of the enrichment protocol. Table S2. Antibodies used in the present study. (PPTX 47 kb) [file 13195_2019_513_MOESM1_ESM.pptx]

## Slide 1
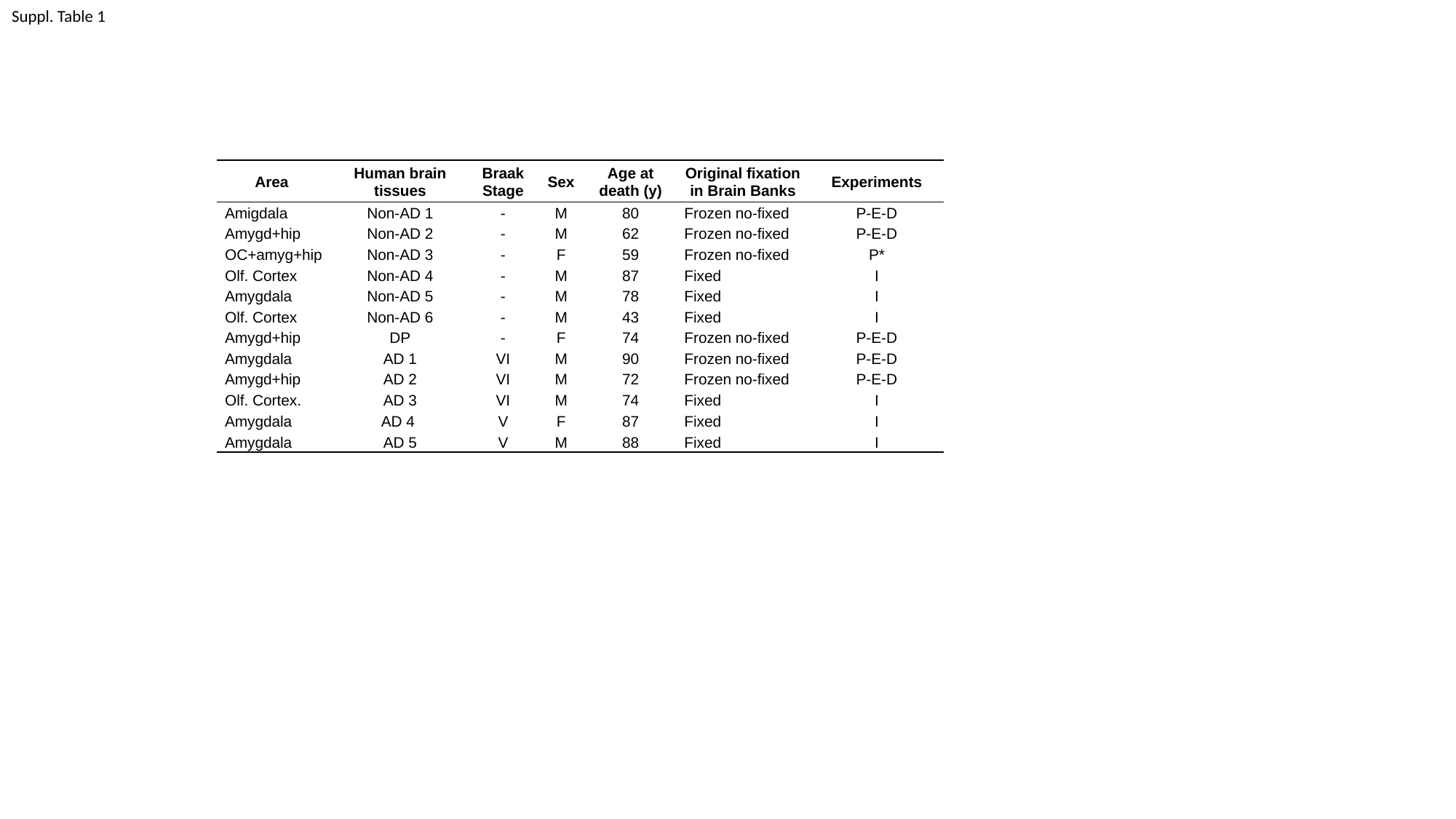

Suppl. Table 1
| Area | Human brain tissues | Braak Stage | Sex | Age at death (y) | Original fixation in Brain Banks | Experiments |
| --- | --- | --- | --- | --- | --- | --- |
| Amigdala | Non-AD 1 | - | M | 80 | Frozen no-fixed | P-E-D |
| Amygd+hip | Non-AD 2 | - | M | 62 | Frozen no-fixed | P-E-D |
| OC+amyg+hip | Non-AD 3 | - | F | 59 | Frozen no-fixed | P\* |
| Olf. Cortex | Non-AD 4 | - | M | 87 | Fixed | I |
| Amygdala | Non-AD 5 | - | M | 78 | Fixed | I |
| Olf. Cortex | Non-AD 6 | - | M | 43 | Fixed | I |
| Amygd+hip | DP | - | F | 74 | Frozen no-fixed | P-E-D |
| Amygdala | AD 1 | VI | M | 90 | Frozen no-fixed | P-E-D |
| Amygd+hip | AD 2 | VI | M | 72 | Frozen no-fixed | P-E-D |
| Olf. Cortex. | AD 3 | VI | M | 74 | Fixed | I |
| Amygdala | AD 4 | V | F | 87 | Fixed | I |
| Amygdala | AD 5 | V | M | 88 | Fixed | I |

## Slide 2
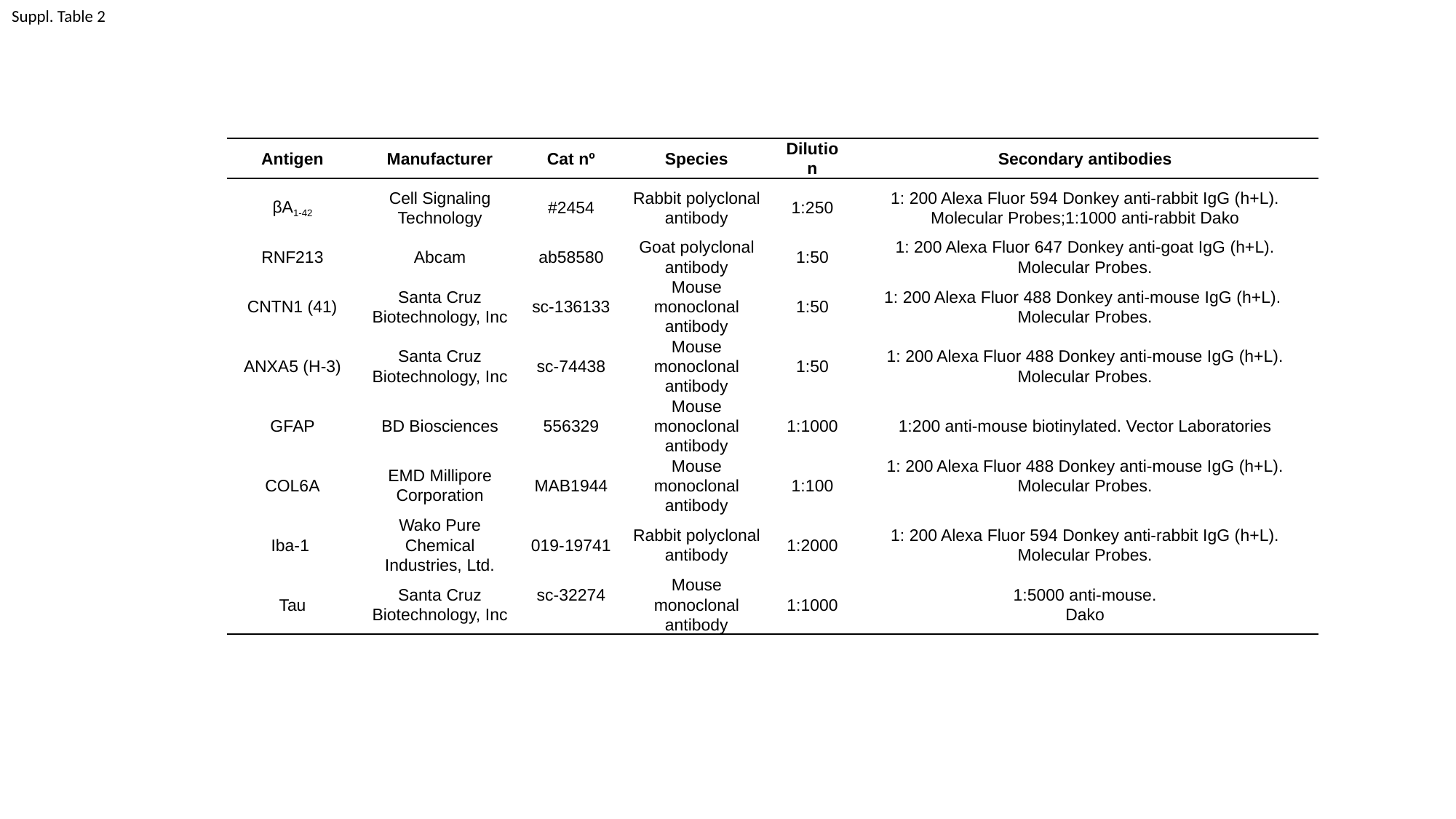

Suppl. Table 2
| Antigen | Manufacturer | Cat nº | Species | Dilution | Secondary antibodies |
| --- | --- | --- | --- | --- | --- |
| βA1-42 | Cell Signaling Technology | #2454 | Rabbit polyclonal antibody | 1:250 | 1: 200 Alexa Fluor 594 Donkey anti-rabbit IgG (h+L). Molecular Probes;1:1000 anti-rabbit Dako |
| RNF213 | Abcam | ab58580 | Goat polyclonal antibody | 1:50 | 1: 200 Alexa Fluor 647 Donkey anti-goat IgG (h+L). Molecular Probes. |
| CNTN1 (41) | Santa Cruz Biotechnology, Inc | sc-136133 | Mouse monoclonal antibody | 1:50 | 1: 200 Alexa Fluor 488 Donkey anti-mouse IgG (h+L). Molecular Probes. |
| ANXA5 (H-3) | Santa Cruz Biotechnology, Inc | sc-74438 | Mouse monoclonal antibody | 1:50 | 1: 200 Alexa Fluor 488 Donkey anti-mouse IgG (h+L). Molecular Probes. |
| GFAP | BD Biosciences | 556329 | Mouse monoclonal antibody | 1:1000 | 1:200 anti-mouse biotinylated. Vector Laboratories |
| COL6A | EMD Millipore Corporation | MAB1944 | Mouse monoclonal antibody | 1:100 | 1: 200 Alexa Fluor 488 Donkey anti-mouse IgG (h+L). Molecular Probes. |
| Iba-1 | Wako Pure Chemical Industries, Ltd. | 019-19741 | Rabbit polyclonal antibody | 1:2000 | 1: 200 Alexa Fluor 594 Donkey anti-rabbit IgG (h+L). Molecular Probes. |
| Tau | Santa Cruz Biotechnology, Inc | sc-32274 | Mouse monoclonal antibody | 1:1000 | 1:5000 anti-mouse. Dako |
