# Supplementary material for: Human amyloid-β enriched extracts: evaluation of in vitro and in vivo internalization and molecular characterization
Source: Alzheimers Res Ther. 2019 Jun 29;11:56. doi: 10.1186/s13195-019-0513-0 (PMC6599264; doi:10.1186/s13195-019-0513-0)
Supplement: Supplementary file 3 — Figure S1. Schemes showing the medial view of the brain and the corresponding levels: A) Coronal sections of olfactory (B) and amygdaloid (C) areas analyzed [Bregma − 5.8 and − 6.7 mm respectively, according to [68]]. The corresponding tissue blocks (D, E) and Nissl-stained, mosaic-reconstructed sections (F, G) are also illustrated. Calibration bars 1 cm. A: amygdala; aic: anterior limb of internal capsule; AONc: anterior olfactory nucleus, cortical part; C: claustrum; cc: corpus callosum; Cd: caudate nucleus; HiH: hippocampal head; ic: internal capsule; LV: lateral ventricle; OlfA: olfactory area; Pir: piriform cortex; PHG: parahippocampal gyrus; Pu: putamen; SG: straight gyrus; TLV: temporal horn of lateral ventricle; un: uncus; Ent: Entorhinal cortex; PRC: perirhinal cortex. Figure S2. Overview of the protocol for the enrichment of Aβ plaques (A). Dot blot of the steps of Aβ plaque enrichment using Aβ1–42 antibody (B–C) and tau antibody (D). Coomassie blue of the steps of Aβ enrichment. AD, Alzheimer disease; Non-AD, non-Alzheimer disease; DP, diffuse plaques; LB, lysis buffer; T, total extract; S, supernatant; P, pellet. Numbers indicate steps in the procedure. Figure S3. Gene ontology overrepresentation study. “p value” is the enrichment p value computed according to the mHG or HG model. “FDR q value” is the correction of the above p value for multiple testing using the Benjamini and Hochberg (1995) method. Figure S4. Double immunofluorescence against Aβ1–42, and ANXA5 (A), and triple immunofluorescence against Aβ1–42, RNF213 and CNTN1 (B). Confocal images of human olfactory cortex sections of human AD samples and non-AD samples to study the distribution of ANXA5 (green, A), RNF213 (green, B), or CNTN1 (purple, B). Immunostaining against Aβ1–42 (red, A and B) was also included to identify Aβ plaques. Nuclei are labeled in blue with DAPI. The arrow indicates nuclei located inside the plaque. Calibration bars 50 μm. (PPTX 11881 kb) [file 13195_2019_513_MOESM2_ESM.pptx]

## Slide 1
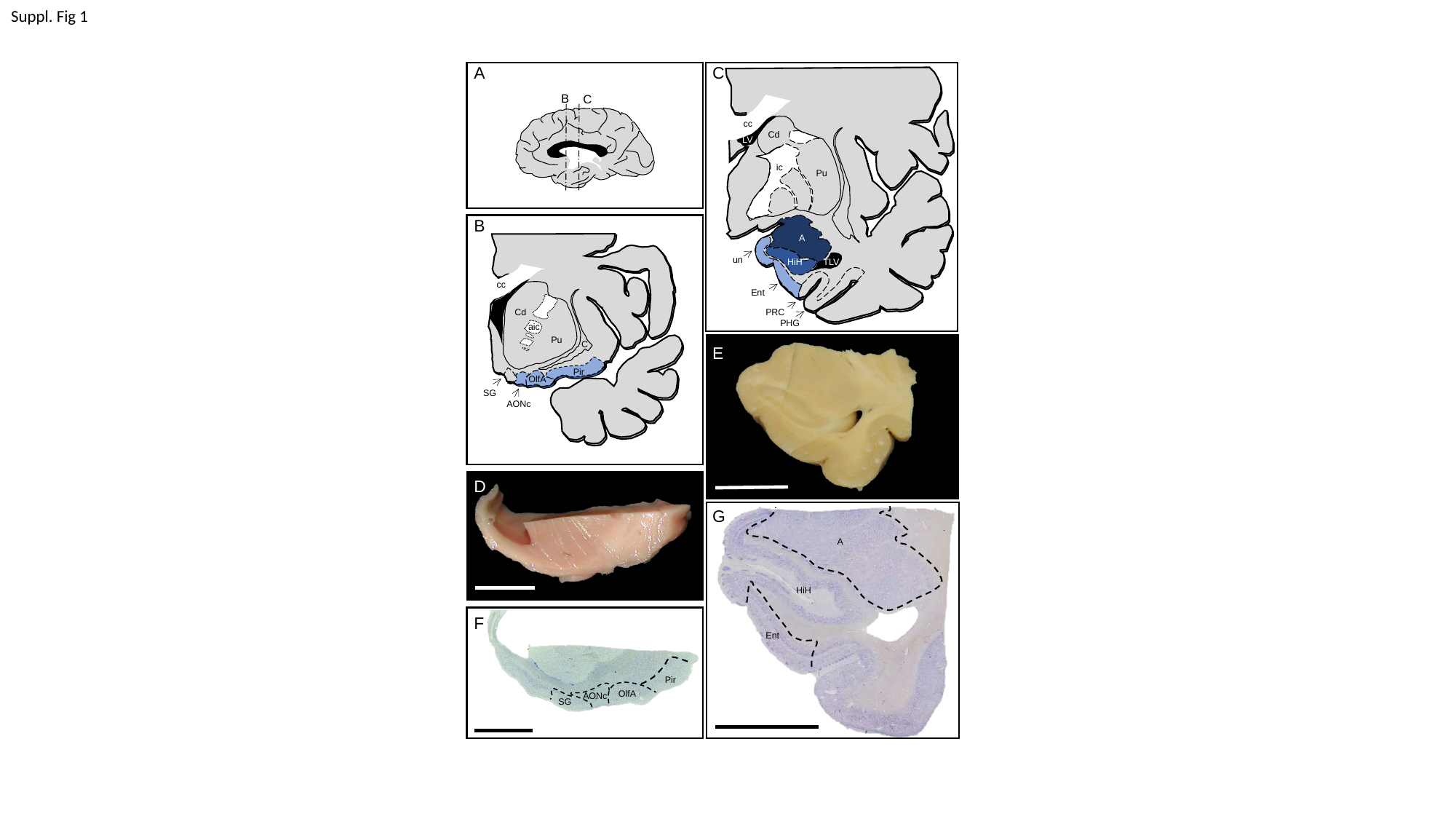

Suppl. Fig 1
A
C
B
C
cc
Cd
LV
ic
Pu
B
A
un
HiH
TLV
cc
Ent
PRC
Cd
PHG
aic
Pu
C
E
Pir
OlfA
SG
AONc
D
G
A
HiH
F
Ent
Pir
OlfA
AONc
SG

## Slide 2
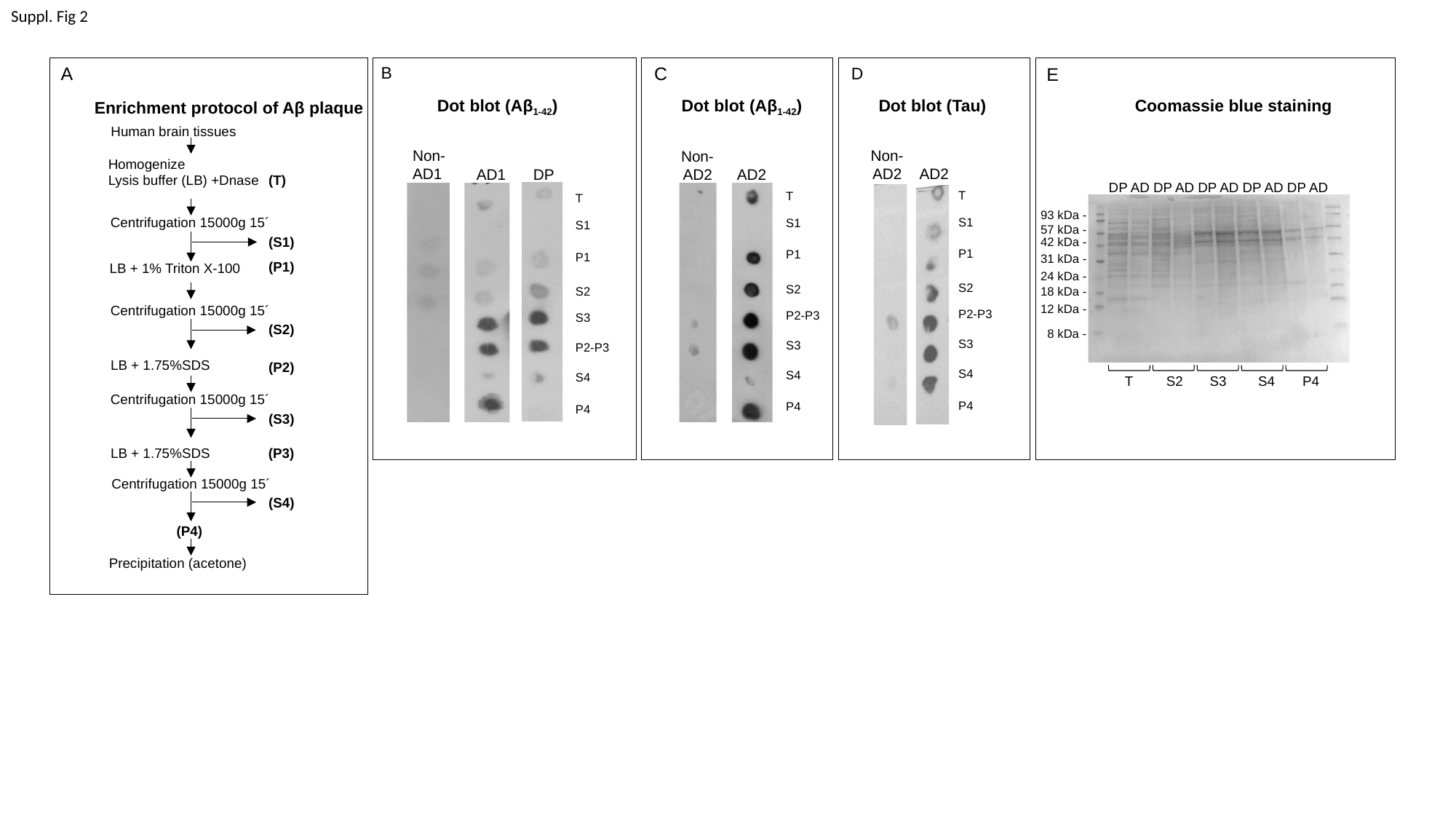

Suppl. Fig 2
 A
B
C
D
E
Coomassie blue staining
 DP AD DP AD DP AD DP AD DP AD
93 kDa -
57 kDa -
42 kDa -
31 kDa -
24 kDa -
18 kDa -
12 kDa -
8 kDa -
T
S2
S3
S4
P4
Dot blot (Aβ1-42)
Dot blot (Aβ1-42)
Dot blot (Tau)
Enrichment protocol of Aβ plaque
Human brain tissues
Non-
AD2
Non-AD1
Non-
AD2
Homogenize
Lysis buffer (LB) +Dnase
AD2
AD1
DP
AD2
(T)
T
T
T
Centrifugation 15000g 15´
S1
S1
S1
(S1)
P1
P1
P1
(P1)
LB + 1% Triton X-100
S2
S2
S2
Centrifugation 15000g 15´
P2-P3
P2-P3
S3
(S2)
S3
S3
P2-P3
LB + 1.75%SDS
(P2)
S4
S4
S4
Centrifugation 15000g 15´
P4
P4
P4
(S3)
LB + 1.75%SDS
 (P3)
Centrifugation 15000g 15´
(S4)
(P4)
Precipitation (acetone)

## Slide 3
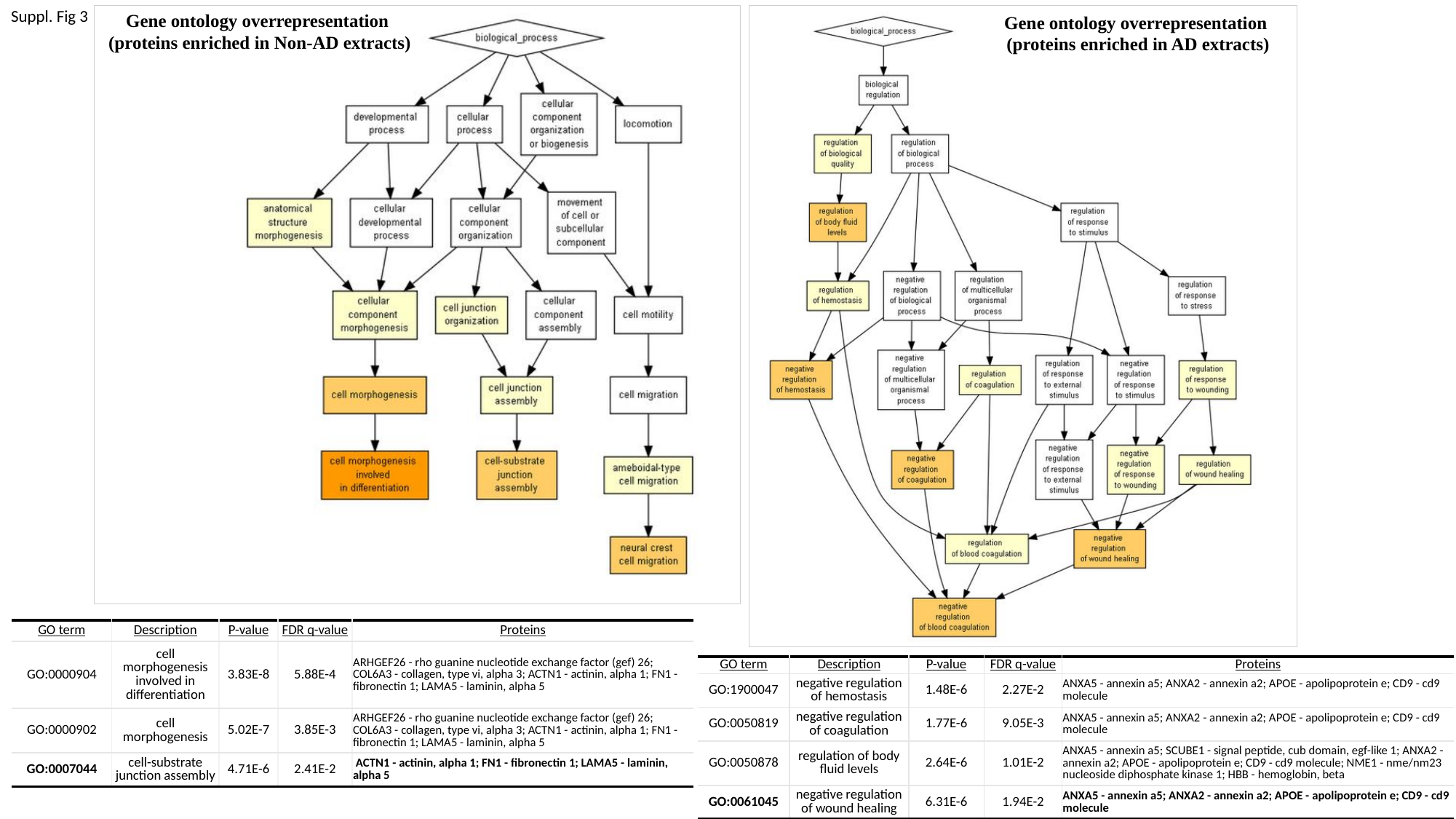

Suppl. Fig 3
Gene ontology overrepresentation
 (proteins enriched in Non-AD extracts)
Gene ontology overrepresentation
 (proteins enriched in AD extracts)
### Chart: AD
| Category |
|---|| GO term | Description | P-value | FDR q-value | Proteins |
| --- | --- | --- | --- | --- |
| GO:0000904 | cell morphogenesis involved in differentiation | 3.83E-8 | 5.88E-4 | ARHGEF26 - rho guanine nucleotide exchange factor (gef) 26; COL6A3 - collagen, type vi, alpha 3; ACTN1 - actinin, alpha 1; FN1 - fibronectin 1; LAMA5 - laminin, alpha 5 |
| GO:0000902 | cell morphogenesis | 5.02E-7 | 3.85E-3 | ARHGEF26 - rho guanine nucleotide exchange factor (gef) 26; COL6A3 - collagen, type vi, alpha 3; ACTN1 - actinin, alpha 1; FN1 - fibronectin 1; LAMA5 - laminin, alpha 5 |
| GO:0007044 | cell-substrate junction assembly | 4.71E-6 | 2.41E-2 | ACTN1 - actinin, alpha 1; FN1 - fibronectin 1; LAMA5 - laminin, alpha 5 |
| GO term | Description | P-value | FDR q-value | Proteins |
| --- | --- | --- | --- | --- |
| GO:1900047 | negative regulation of hemostasis | 1.48E-6 | 2.27E-2 | ANXA5 - annexin a5; ANXA2 - annexin a2; APOE - apolipoprotein e; CD9 - cd9 molecule |
| GO:0050819 | negative regulation of coagulation | 1.77E-6 | 9.05E-3 | ANXA5 - annexin a5; ANXA2 - annexin a2; APOE - apolipoprotein e; CD9 - cd9 molecule |
| GO:0050878 | regulation of body fluid levels | 2.64E-6 | 1.01E-2 | ANXA5 - annexin a5; SCUBE1 - signal peptide, cub domain, egf-like 1; ANXA2 - annexin a2; APOE - apolipoprotein e; CD9 - cd9 molecule; NME1 - nme/nm23 nucleoside diphosphate kinase 1; HBB - hemoglobin, beta |
| GO:0061045 | negative regulation of wound healing | 6.31E-6 | 1.94E-2 | ANXA5 - annexin a5; ANXA2 - annexin a2; APOE - apolipoprotein e; CD9 - cd9 molecule |

## Slide 4
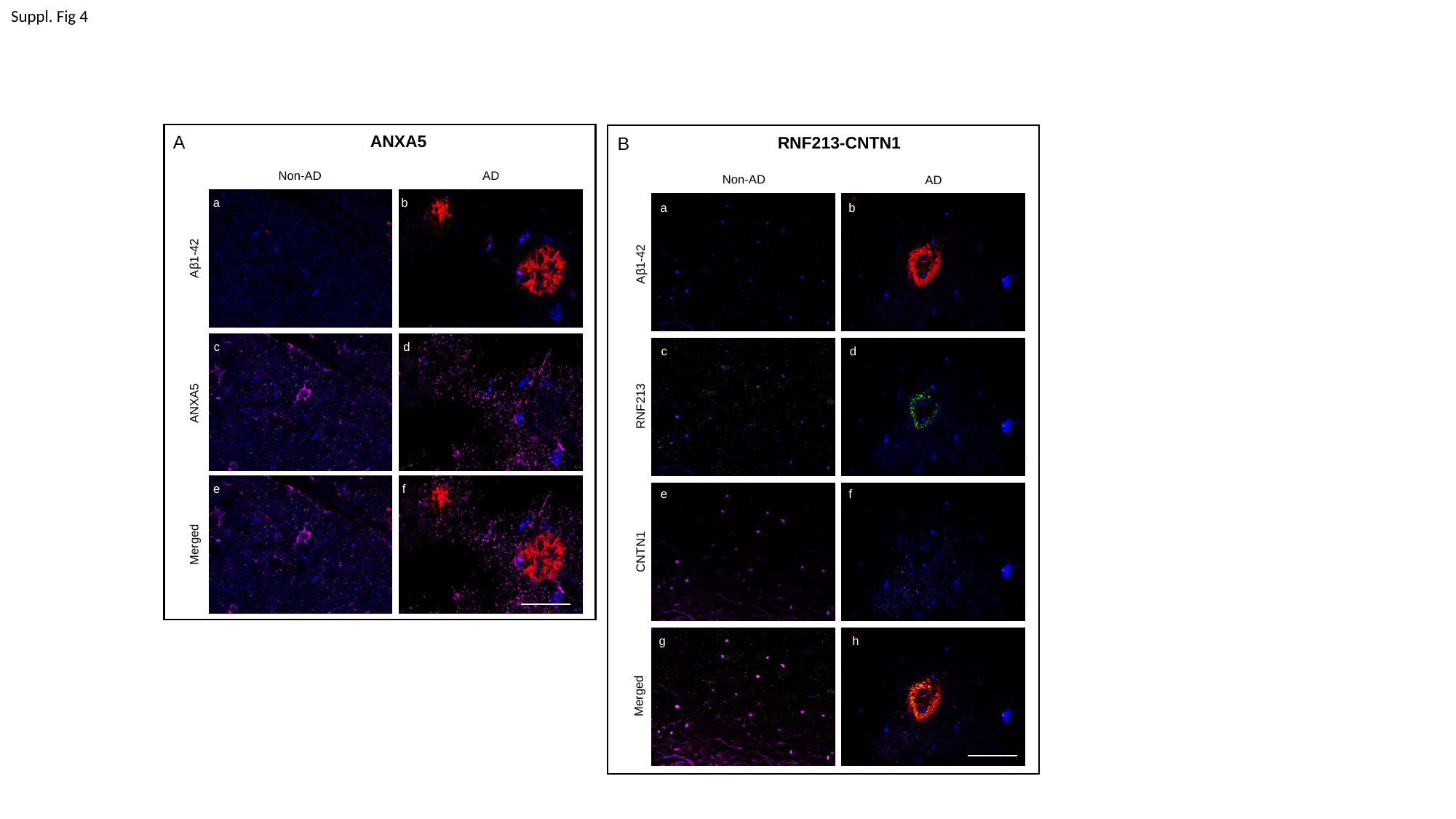

Suppl. Fig 4
A
ANXA5
B
RNF213-CNTN1
Non-AD
AD
Non-AD
AD
E
a
b
M
a
b
Aβ1-42
Aβ1-42
c
d
c
d
ANXA5
RNF213
e
f
e
f
Merged
CNTN1
g
h
Merged
